# Non-Canonical Kinases and Substrates in Cancer Progression

**DOI:** 10.3390/cancers13071628

**Published:** 2021-04-01

**Authors:** Francisco M. Vega

**Affiliations:** 1 Departamento de Biología Celular, Facultad de Biología, Universidad de Sevilla, 41012 Seville, Spain; fmvega@us.es; 2 Instituto de Biomedicina de Sevilla (IBiS), Hospital Universitario Virgen del Rocío, CSIC, Universidad de Sevilla, 41013 Seville, Spain

Cellular protein kinases remain the target of choice when the intention is to intervene in a particular signaling pathway leading to cancer progression. Their reversible mode of action, tight regulation, and molecular structure allows for the design of specific inhibitors. However, from the more than 500 protein kinases identified, many potentially involved in important cancer-related signaling cascades, only a few have received most of the attention over the years. This is either due to their central role on essential processes, the facility of intervention, or their early discovery. This Special Issue tried to offer new insights into some of the lesser-known human protein kinases, their substrates, regulation, and involvement in cancer progression. The published articles offer good examples of the important cellular functions that the so-called non-canonical kinases develop and their contribution to cancer. They also discuss some of the therapeutical opportunities and challenges.

The vaccinia-related kinase (VRK) proteins are structurally related to the casein kinase family and the viral protein B1R. This family of protein kinases has revealed itself as an important mediator of tumor progression and cell proliferation. Although a role for VRK1 in normal cell division is granted, the high expression and activity of this protein in some cancers, including now pediatric neuroblastoma, is essential for malignization [1]. VRK1 synergizes with other oncogenes to drive cancer progression, notably with NMYC in neuroblastoma. Increasingly, data point to their possible use as a prognostic marker and therapeutical target. However, the complexity of its regulation and substrates makes detailed molecular studies about this chromatin remodeling enzyme necessary. Here, García-González et al. also report a role for VRK1 in chromatin acetylation leading to DNA damage response, indicating that VRK1 could also be involved in increased genome instability [2]. It is now clear that the expression of some of these non-canonical kinases could be incorporated as new prognostic markers or biomarkers of therapy response in various cancers. Another example is offered by the study of the protein kinase R (PKR) and its regulator, the non-coding pre-mir-nc886, in colorectal cancer responses after chemotherapy [3].

Another family of pleiotropic protein kinases associated with cancer is dual-specificity tyrosine-regulated kinases (DYRK). Their molecular functions leading to tumor progression are discussed in another contribution to this Special Issue [4]. Interestingly, small DYRK inhibitors have been developed, offering new therapeutic avenues. Recent evidence has established the importance of the tumor microenvironment for cancer progression and there is a need to understand the signaling pathways mediating the response and adaptation of cancer cells to external stimuli. In their contribution to the Special Issue, Reglero et al. describe how a GRK2-dependent phosphorylation can modulate Hypoxia Inducible Factor (HIF)-dependent responses to hypoxia in cancer cells [5].

Specific tyrosine kinase inhibitors are being used with success in the treatment of some types of cancer. In addition to major signaling players, tyrosine kinase inhibitors also offer specificity against other tyrosine kinases. A revision of their use and targets could offer new opportunities for the treatment of tumors with poor prognosis and few therapeutic tools available [6]. For example, the inhibitor sunitinib is used as an antiangiogenic inhibitor, principally for its action against the VEGF receptors, VEGFR1 and VEGFR2, and the PDGF receptor. Sunitinib also inhibits other protein kinases such as Fms Related Receptor Tyrosine Kinase 3 (FLT3) or KIT. These kinases seem to be the main targets in glioblastoma and, when combined with a cytotoxic therapy such as boron neutron capture, offer a good response in these difficult-to-treat tumors [7]. This is an example in which well-known inhibitors use could be expanded for their action over non-canonical kinases. In most cases, the attention, even in widely studied protein kinases, has been focused on kinase activity, ignoring other protein domains with potentially important molecular functions. The Src tyrosine kinase was one of the first oncogenic kinases discovered. However, much less attention has been paid to the adapter domains SH2 and SH3 in the molecule. The results presented in this Special Issue by Mayoral-Varo et al. explore the functionality of these domains in breast cancer cells, suggesting a synergistic effect between their inhibition and the inhibition of the kinase activity [8].

In summary, this Special Issue of *Cancers* shows good examples of the functionality and therapeutic values that the study of non-canonical kinases or domains, their substrates, and inhibition can offer. With research on these lesser-known proteins, we will expand not only our knowledge about cancer progression, but our possibilities to halt it.
